# Effectiveness of a multimodal physiotherapy program in fighter pilots with flight‐related neck pain: A randomized controlled trial

**DOI:** 10.1002/pmrj.13399

**Published:** 2025-05-23

**Authors:** Carlos Fernández‐Morales, Manuel Albornoz‐Cabello, María de los Ángeles Cardero‐Durán, Juan Manuel Moreno‐Vázquez, Luis Espejo‐Antúnez

**Affiliations:** ^1^ Department of Medical‐Surgical Therapy. Faculty of Medicine and Health Sciences University of Extremadura Badajoz Spain; ^2^ Department of Physiotherapy University of Seville Seville Spain; ^3^ Department of Biomedical Sciences, Faculty of Medicine and Health Sciences University of Extremadura Badajoz Spain

## Abstract

**Background:**

Flight‐related neck pain constitutes a clinical entity related to the performance, occupational health, and flight safety of fighter pilots.

**Objective:**

To investigate whether a multimodal physiotherapy program combining supervised motor control exercises with laser‐guided feedback and interferential current therapy with electromassage improves cervical pain and function outcomes in fighter pilots with flight‐related neck pain compared to their usual operational routines.

**Design:**

Thirty‐one pilots participated in the study, divided into two groups: intervention group (*n* = 14), who received eight sessions of a multimodal physiotherapy program (twice a week for 4 weeks), and a control group (*n* = 17), who did not receive any intervention and maintained their usual operational routines. Primary outcome measures were perceived pain intensity (Numeric Pain Rating Scale) and Joint Position Sense Error. The secondary outcome measures were neck disability (Neck Disability Index), cervical range of motion, and pressure pain threshold. Measurements were taken at baseline and immediately after treatment. An intention‐to‐treat analysis was carried out. To quantify the effect size of the interventions, the relative risk, the absolute and relative risk reduction, and the number needed to treat were calculated.

**Results:**

Statistically significant changes were observed between groups in favor of the intervention group in both primary outcomes measures: Numeric Pain Rating Scale (mean difference: 2.5 [1.5–3.5]; *p* < .001; d = 0.7); Joint Position Sense Error (mean difference: 2 [1.5–2.5]; *p* < .001; d = 0.8), and secondary outcomes measures (*p* < .05), with large effect sizes (d ≥ 0.8) in cervical range of motion and pressure pain threshold and moderate (d ≥ 0.6) in Neck Disability Index. In the analysis for treatment benefit, the number needed to treat was 2 (95% confidence interval, 2–3, *p* < .001) for neck pain and proprioceptive acuity.

**Conclusions:**

A multimodal physiotherapy program based on supervised exercises with laser‐guided feedback and interferential current therapy improves the symptoms and cervical function of fighter pilots with flight‐related neck pain.

## INTRODUCTION

Neck pain represents one of the most common musculoskeletal conditions, being a major health problem.^1^ It is associated with high socioeconomic impact,^1^ partly because of its peak prevalence among the working population, between 30% and 50%.^2^ This prevalence increases significantly in fighter pilots, with rates ranging from 66% to 83% across different air forces.^3^ Concretely, according to North Atlantic Treaty Organization (NATO), flight‐related neck pain (FRNP) has been defined as a clinical entity specific to military pilots.^4^ It refers to the significant neck pain that occurs during or within 48 hours after the flight. It does not refer to pain that is obviously due to other activities or causes. Underlying mechanisms of FRNP persistence, recurrence, and progression are not clear, but they could be associated with some occupational factors such as prolonged and repetitive exposure to high G‐forces, awkward postures during flight operations, and the cognitive and physical demands required for flight safety, including helmet‐mounted equipment and mission intensity.^3^, ^4^ In this sense, Espejo et al.^3^ have identified cervical mobility deficit in fighter pilots associated with FRNP. Additionally, proprioceptive impairments are also relevant factors influencing motor control of the head.^3^, ^5^, ^6^

Current evidence‐based guidelines for the management of neck pain highlight the importance of multimodal physiotherapy programs based on the interaction between techniques.^2^, ^7^ Manual therapy (eg, with or without impulse, muscle release technique, soft tissues mobilization) and therapeutic exercise (supervised or nonsupervised) are the most recommended treatments.^1^, ^4^ However, evidence is insufficient when it comes to physiotherapy‐based treatments for managing FRNP, which differs from other types of neck pain due to the combination of high mechanical loads and cognitive demands associated with flight combat, requiring targeted therapeutic approaches.^3^, ^7^ Recently, NATO has recommended transcutaneous electrical stimulation as one of the first‐choice treatment in fighter pilots with FRNP.^4^ However, unlike clinical practice guidelines, NATO's recommendations provide a broader framework without establishing a specific hierarchy of preferred treatments.

There are various modalities within transcutaneous electrical stimulation, including interferential current therapy (ICT). Previous studies have reported clinical benefits of combining interferential current with therapeutic exercise for managing neck pain,^8^ although some controversial aspects regarding its efficacy on cervical range of motion (CRoM) remain. Considering these recommendations, recent approaches have explored innovative procedures, such as interferential current electro‐massage, which has shown effectiveness in specific populations, including fighter pilots.^9^ However, these techniques have not been combined into an integrated multimodal program specifically designed for fighter pilots with FRNP.

On the other hand, previous studies carried out therapeutic exercise to treat neck pain in military workers. Heng et al.^10^ analyzed the benefits of cervical proprioceptive training and motor learning‐based exercises versus protocols that only included strength or endurance exercises in reducing pain. The development of rehabilitation technologies has reported differences between types of exercise according to whether the exercise requires the individual to focus on their body while moving a body region (exercise with an internal locus of movement control) or to focus on clues provided by the environment, with the effect being achieved after movement has been performed (exercise with an external locus such as a laser pointer).^11^ The clinical utility has been explained as facilitating the establishment of effective neural connections that optimize exercise performance.^12^

Incorporating research findings into clinical practice requires an understanding of their clinical relevance.^13^ Statistical tests such as relative risk (RR), absolute risk reduction (ARR) and relative risk reduction (RRR), and number needed to treat (NNT) provide valuable insights for guiding clinical decisions.^14^ However, these metrics are rarely explored in the context of aviation medicine, particularly in studies involving fighter pilots with FRNP.

There is no clear consensus about which is the most effective approach for fighter pilots with FRNP. Current evidence suggests that active approaches, such as motor control exercises adapted to the occupational demands of fighter pilots, could address the specific cervical dysfunctions caused by flight‐related mechanical stressors.^15^ This study hypothesizes that a multimodal physiotherapy program combining supervised motor control exercises with laser‐guided feedback (ELGF) and ICT would improve cervical pain and function outcomes in fighter pilots with FRNP compared to their usual routines. The aim of this study was to evaluate the effectiveness of this multimodal physiotherapy program (ICT with electro‐massage combined with ELGF) in improving cervical pain and function outcomes in comparison with the usual routines of fighter pilots.

## MATERIALS AND METHODS

### Study design

We performed a randomized single‐blind pilot trial (NCT05541848) with concealed allocation, intention‐to‐treat analysis and the assessor and statistic researcher being blinded to treatment allocation. The study was carried out in compliance with the recommendations of the Consolidated Standards of Reporting Trials statements.^16^ The present study was conducted following the Declaration of Helsinki, and it was approved by the Ethical Research Committee of the University of Extremadura (54/2020).

### Participants

**FIGURE 1:**
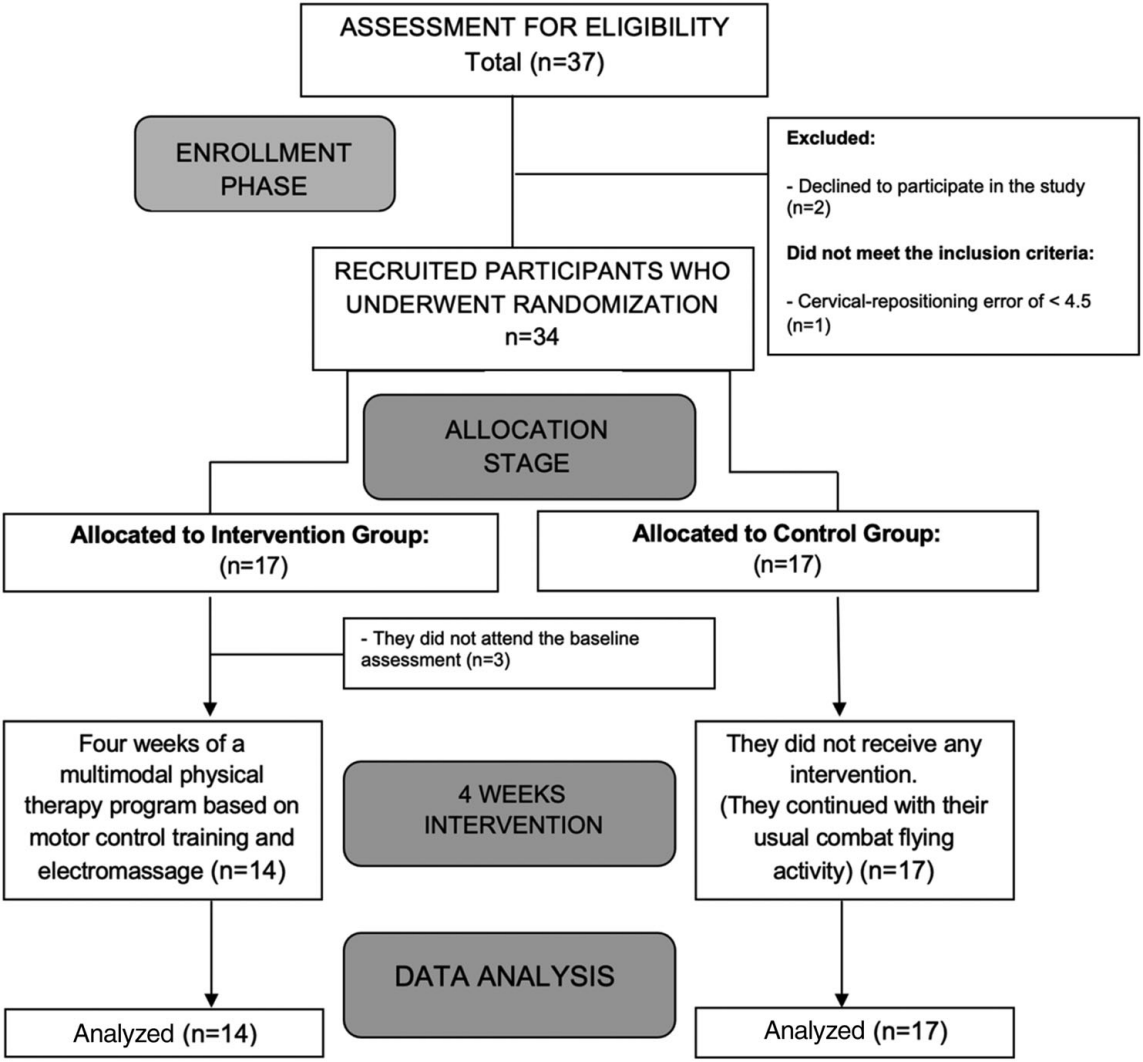
Flow chart diagram of the progress of patients through the study phases.

Following a convenience sampling, recruitment took place from September 2022 to December 2022 at Talavera Air Base in southern Spain. An initial, potentially eligible sample of 37 fighter pilots were recruited. The inclusion criteria were (1) fighter pilots who, at the time of the assessment, were either an instructor or a student attached to the 23th Wing of Talavera Air Base, Spanish Air Force, Badajoz; (2) fighter pilots diagnosed with FRNP according to the International Classification proposed by a NATO panel of experts^4^; (3) a minimum perceived pain of 3/10 on the Numeric Pain Rating Scale (NPRS) in the early‐morning assessment within 48 hours of the last flight; and (4) scores of ≥5 points on the Neck Disability Index (NDI) and cervical movement control dysfunction (cervical‐repositioning error of ≥4.5°).^17^ The exclusion criteria were (1) Personal Psychological Apprehension Scale score >37.5^18^; (2) contraindication for electrical stimulation; (3) having received physiotherapy or any other routine medical care 6 weeks prior to data collection; (4) any regular use of medications including opioids, antidepressants, benzodiazepines, anti‐inflammatory drugs, and beta blockers, 2 weeks before participating in this study; and (5) being involved in ongoing medical–legal conflicts. The total sample consisted of 31 fighter pilots (Figure 1). Figure 1 provides a flow diagram of participant recruitment during the study.

### Randomization

An external website (http://www.randomization.com) was used to complete the group allocation. Participants were randomly allocated (using block randomization, 1:1) into one of the two groups created: the intervention group (IG) and the control group (CG). The randomization was performed by an external assistant. Two researchers were in charge of the study procedure. A blinded researcher collected the measurements at baseline and immediately after the treatment. The other researcher was in charge of implementing the intervention in both groups. Both were physiotherapists with more than 15 years of experience.

### Assessment

All data were collected in the same room, at a temperature of 22–25°C. For the assessment of fighter pilots with FRNP, the recommendations established in clinical practice guidelines for the assessment of participants with neck pain were followed.^1^ Following the definition of FRNP,^4^ assessments were conducted within 48 hours after the last flight, which corresponded to routine operational missions. Additionally, the posttreatment evaluation was carried out 48 hours after the last treatment session. Recruitment and inclusion were conducted on separate days. All assessments were performed in the same order and under the same environmental conditions for all participants.

#### Primary outcomes

##### Numeric pain rating scale

The NPRS is a 11‐point numeric rating scale, where 0 denotes “no pain” and 10 denotes “the maximum bearable pain.” The minimum clinically important difference (MCID) for this tool has been established at 1.5 points, and the minimum detectable change (MDC) is 2.6 points in individuals with neck pain. The NPRS is a valid scale with moderate test–retest reliability in this population (intraclass correlation coefficient [ICC]: 0.76 [95% confidence interval (CI)] 0.58–0.93]).^8^, ^19^

##### Cervical joint position sense error

The Joint Position Sense Error (JPSE) assesses the ability to reposition the head in its natural posture. It is a test of cervical proprioception.^17^ The Motion Guidance Clinic Kit (Motion Guidance LLC, Denver, CO, USA), a visual feedback device, was used. Participants sat on a chair with their heads in a neutral position and a laser pointer attached to their forehead. A target was placed 90 cm away and aligned with the laser. Participants were instructed to memorize the initial position with their eyes open, close their eyes, rotate their head to the right, and then return to the starting position as accurately as possible. Six trials were performed, with the head manually repositioned between each attempt. The average error was calculated, and a mean error greater than 4.5° was considered abnormal.^17^ JPSE is a valid and reliable test in the clinical evaluation of patients with neck pain (ICC, [95% CI, 0.30–0.78]) and the MDC ranges between 0.44° and 0.63°.^20^

#### Secondary outcomes

##### Neck disability index

This outcome measure was evaluated using the validated Spanish version of the NDI.^20^ NDI has been shown to be valid and reliable for measuring pain and cervical disability (Cronbach Alpha: 0.944; ICC, 0.88 [95% CI, 0.80–0.93).^21^ The MCID has been reported at 9.5 points and MDC at 9.8 points.^22^

##### Cervical range of motion

Goniometry is a valid and reliable measuring instrument used in the cervical region.^23^ The evaluator measured the sagittal (flexion/extension), frontal (right/left lateral flexion), and transversal plane (right/left rotation) using an individual head goniometer (Enraf‐Nonius BV, Rotterdam, The Netherlands) as indicated by Nagai et al.^24^ Each physiological movement was performed three times with 30 seconds of rest.^3^ The values obtained were referenced with the results reported by Kauther et al.^25^ In people with neck pain, the SE of the device ranges from 2.9 (left rotation) to 4.1° (flexion). The MDC ranges between 5.9° (right side bending) and 9.6° (flexion).^23^

##### Pressure pain threshold

Pressure pain threshold (PPT) of myofascial trigger points of the sternocleidomastoid muscle, upper trapezius, and levator scapulae muscle were evaluated with a mechanical pressure algometer (Baseline Fabrication Enterprise. Inc. White Plains, NY), coinciding with the characteristics indicated by Lange et al.^5^ Moderate‐high reliability has been reported, with ICC 0.91 (95% CI, 0.82–0.97). The MCID is 1.5 kg/cm^2^.^26^

Three PPTs measurements were taken with a 2‐minute rest interval between each trial. The mean of the three trials was calculated and used for the analysis.

### Intervention

The participants in the intervention group (*n* = 14) followed a program of supervised ELGF (Supplementary material S1). Afterwards, they received an intervention based on ICT, which was applied by means of the so‐called electro‐massage^27^ (Supplementary material S2). They completed a total of eight sessions in 4 weeks. The multimodal program was performed in all pilots within 48 hours after the last flight. The participants in the control group (*n* = 17) did not receive any intervention. During this phase, both groups maintained the same operational routines, including flight hours and workload. They were asked not to take medication or to seek alternative treatments. This design was chosen to observe the natural course of the condition and to evaluate the real‐world impact of the multimodal program, ensuring its potential feasibility and applicability within the operational routines of the airbase. The intervention took place at Talavera Air Base (Spain).

#### Interferential current therapy with electro‐massage

ICT with electro‐massage simultaneously combines manual therapy (massage) with interferential current,^27^ involving a 15‐minute treatment performed in the cervical region. We used a current bipolar mode, using a carrier frequency of 4000 Hz at constant voltage and an amplitude‐modulated frequency of 100 Hz (Sonopuls 692; Enraf‐Nonius BV, Rotterdam, The Netherlands). Unlike other modalities of transcutaneous electrical stimulation, ICT allows for the targeting of deep tissues, increasing the temperature of the collagen matrix.^28^ The therapist applies manual soft tissue therapy while administering ICT through moistened sponges, focusing on the neck, shoulder, and scapular areas. The protocol includes various techniques such as superficial strokes, deep sliding movements, kneading of the upper trapezius, and gentle stretching of cervical muscles.

#### Cervical supervised exercises with laser‐guided feedback

Cervical supervised ELGF use external feedback to facilitate automatic control and retention of movement patterns.^12^, ^29^ The Motion Guidance Clinician Kit was used, consisting of a panel and laser guide positioned on the forehead for precise execution of cervical movements. The speed of movement was adjusted depending on the participant, as were the length of hold, range of movement (eg, half‐range or three quarters range), and exercise position. The program included progressively challenging exercises performed in seated and standing positions. taking an average of 14 minutes to complete, adhering to the recommendations of the Consensus on Exercise Reporting Template^30^ and Template for Intervention Description and Replication^31^ statements. During the sessions, adherence was assumed to be 100% if participants completed all eight scheduled sessions under the established conditions.

### Sample size

Sample size calculation for detecting changes in the primary outcome (NPRS scale and JPSE) was performed using G*power 3.1 software. Considering an effect size (F‐test) of 0.27 for analysis of variance: repeated measures, within‐between interaction groups differences, an alpha level of .05, and a power of 80%, a total sample size of 30 participants was estimated. To account for potential dropouts, the sample was inflated by 10%, resulting in a final target sample size of 34. This calculation indicated that a sample size of 17 participants per group was required to achieve a 95% CI with 80% power, assuming a bilateral significance level of .05.

### Statistical analysis

Descriptive analysis was conducted for each variable. Normality tests were performed on the variables analyzed using the Shapiro‐Wilk test. The nonparametric Wilcoxon signed‐rank test (when normality was not admissible) or Student's *t*‐test for related samples (under normality) was used to compare each variable within groups before and during the intervention. Subsequently, the Student's *t*‐test or Welch's test (under normality) or the nonparametric Mann‐Whitney or Wilcoxon test (when normality was not acceptable) was applied to compare the groups. Mean ± SD and median (interquartile range) were used to report the data. Additionally, Cohen's d coefficient or Rosenthal's r was calculated to determine effect size, with values >0.8 considered high, 0.5 considered moderate, and <0.2 considered low. To further analyze treatment benefit, the RR, ARR, RRR, and NNT were calculated, along with their 95% CI for the primary outcomes. RR measures the ratio of the probability of an event occurring in the intervention group to the probability of the same event occurring in the control group.^8^ ARR measures how much lower the probability of the event occurring is in the intervention group compared to the control group. It is calculated as the absolute difference in event proportions between the control and intervention groups, and RRR measures the reduction in risk in the intervention group relative to the control group. It is calculated as the difference in event proportions between the control and intervention groups, divided by the event proportion in the control group.^14^ Finally, the NNT represents the number of patients who need to receive the intervention for one additional patient to benefit, compared to a control group.^32^ To confirm that there are no other variables that could influence this result, simple correlations between the statistically significant results (NPRS and JPS) and the sociodemographic variables of the groups at the beginning of the study were analyzed using Pearson's test or Spearman's Rho test. Statistical significance was set at *p* < .05. Data analysis was carried out using SPSS version 26.0 (SPSS Inc., Chicago, IL, USA).

## RESULTS

Table 1 lists the baseline clinical and demographic features of participants. There were no significant differences in the analysis between groups at baseline for any of the variables (all, *p* > .05). Three participants dropped out of the intervention group due to work‐related demands. No adverse events were recorded.

**TABLE 1:** Baseline characteristics of participants (mean ± SD or [95% confidence level]; and median [IQR]).

	Total sample (*N* = 31)	Intervention group (*N* = 14)	Control group (*N* = 17)	e*
Age (years)	25 ± 6.4 22 [22–30]	24 ± 5.5 22 [22–24]	26 ± 7.1 22 [21.5–30]	.49
Height (cm)	178 ± 6.5 178 [174–184]	179 ± 5.8 180 [176.5–184.3]	178 ± 7.1 176 [173–184]	.49
Weight (kg)	76.2 ± 8.4 75 [71–84]	77.7 ± 6.8 77 [72.8–84.5]	75.0 ± 9.6 72 [70–83]	.38
BMI (kg/m^2^)	23.8 ± 1.9 23.5 [22.7–25.4]	24.0 ± 1.4 23.7 [22.9–25.4]	23.6 ± 2.2 23.3 [22.2–25.8]	.56
Number of flight hours/week	4.4 ± 1.5 4 [3–5]	3.8 ± 1.2 3 [3–5]	4.8 ± 1.6 4.5 [3.5–7]	.06
Number of hours of exercise/week	3.6 ± 1.7 3 [2.5–4]	3.9 ± 1.4 3.5 [3–5]	3.3 ± 1.9 3 [2–5]	.31
NPRS (0–10)	5.5 ± 1.7 6 [4–7]	6.1 ± 1.8 6.5 [4.8–8]	5.1 ± 1.5 5 [4–6.5]	.09
JPSE (°)	5.4 ± 0.7 5.5 [4.8–5.8]	5.2 ± 0.6 5.3 [4.7–5.6]	5.6 ± 0.7 5.8 [5.1–6.1]	.12
NDI (0–50)	19 ± 5.1 18 [15–22]	21 ± 5.6 19 [16.5–25.5]	18 ± 4.4 18 [15–21]	.16
CRoM flexion (°)	36 ± 4.5 36 [32–40]	36 ± 4.6 35.5 [32–40]	36 ± 4.5 37 [31.5–40]	.96
CRoM extension (°)	41 ± 8.5 40 [32–49]	42 ± 9.8 42.5 [31.5–50]	40 ± 7.4 40 [33–47]	.42
CRoM lateral flexion left (°)	44 ± 9.4 45 [40–49]	44 ± 12.4 41 [32–56.3]	44 ± 6.1 45 [40–48]	.95
CRoM lateral flexion right (°)	45 ± 10.2 45 [37–51]	46 ± 13.6 47.5 [34.3–58.5]	43 ± 6.4 44 [39–48]	.42
CRoM rotation left (°)	43 ± 6.8 43 [38–50]	42 ± 7.2 47.5 [34.3–58.5]	44 ± 6.5 45 [38.5–51]	.42
CRoM rotation right (°)	44 ± 5.4 44 [40–46]	44 ± 3.9 45 [40.8–45.3]	44 ± 6.5 42 [39.5–50]	.85
PPT trapezius left (kg/cm^2^)	2.8 ± 0.6 2.6 [2.3–3.5]	2.6 ± 0.6 2.7 [2.4–3.4]	3.0 ± 0.6 3 [3.5–2.6]	.11
PPT trapezius right (kg/cm^2^)	2.9 ± 0.4 3 [2.6–3.4]	2.9 ± 0.5 3 [2.6–3.5]	2.9 ± 0.3 2.9 [2.6–3.4]	.98
PPT SCM left (kg/cm^2^)	1.4 ± 0.3 1.3 [1.2–1.7]	1.3 ± 0.3 1.3 [1.2–1.6]	1.5 ± 0.4 1.3 [1.3–1.9]	.17
PPT SCM right (kg/cm^2^)	1.4 ± 0.3 1.4 [1.2–18]	1.5 ± 0.3 1.5 [1.2–1.9]	1.4 ± 0.3 1.3 [1.2–1.7]	.42
PPT levator scapulae left (kg/cm^2^)	3.2 ± 0.6 3.4 [2.6–3.8]	3.3 ± 0.6 3.5 [2.7–3.8]	3.2 ± 0.5 3.1 [2.8–3.7]	.75
PPT levator scapulae right (kg/cm^2^)	3.1 ± 0.5 3.1 [2.8–3.5]	3.1 ± 0.4 3.1 [2.8–3.6]	3.2 ± 0.6 3 [2.8–3.7]	.42

*Note*: Data are reported as mean ± SD and median [IQR].

Abbreviations: BMI, body mass index; CRoM, cervical range of motion; IQR, interquartile range; JPSE, Joint Position Sense Error; NDI, Neck Disability Index; NPRS, Numeric Pain Rating Scale; PPT, pressure pain threshold; SCM, sternocleidomastoid.

* Between‐group statistical significance.

Table 2 shows the baseline and postintervention scores and the mean differences between and within groups for all the variables subject to study. In comparison to the baseline values, IG showed significant changes (*p* < .001) and moderated effect size (d ≥ 0.6) in the NDI, PPT, and CRoM and large effect sizes (d ≥ 0.8) in NPRS and JPSE.

**TABLE 2:** Baseline, postintervention, and mean score changes (mean ± SD or [95% confidence level]; and median [IQR]).

		Baseline	Post intervention	Pre/post differences	Between‐group mean changes post intervention
NPRS (0–10)	Intervention	6.1 ± 1.8 [5.0–7.1] 6.5 [4.8–8.0]	1.6 ± 1.6 [7.0–2.6] 1.0 [0.0–3.0]	4.5 [3.3–5.5] *p* < .001** (0.8)^△^	2.5 [1.5–3.5] *p* < .001^††^ (0.7)^△△^
	Control	5.1 ± 1.5 [4.3–5.8] 5.0 [4.0–6.5]	4.1 ± 1.0 [3.6–4.6] 4.0 [3.0–5.0]	1.0 [0.5–1.4] *p* = .004* (0.7)^△△^	
JPSE (°)	Intervention	5.2 ± 0.6 [4.8–5.5] 5.3 [4.7–5.6]	2.4 ± 0.5 [2.1–2.7] 2.5 [1.9–2.7]	2.8 [2.6–3.1] *p* < .001** (0.9)^△△^	2.0 [1.5–2.5] *p* < .001^††^ (0.8)^△^
	Control	5.6 ± 0.7 [5.2–6.0] 5.8 [5.1–6.1]	4.4 ± 0.8 [3.9–4.8] 4.5 [3.8–4.8]	1.2 [0.9–1.5] *p* < .001** (0.6)^△^	
NDI (0–50)	Intervention	21 ± 5.6 [17–24] 19 [16.5–25.5]	12 ± 3.3 [10–14] 11 [10–13]	9 [5.6–11.6] *p* < .001** (0.7)^△^	5 [2.3–7.5] *p* < .001^††^ (0.6)^△△^
	Control	18 ± 4.4 [16–20] 18 [15–21]	17 ± 3.6 [15–19] 17 [14.5–19]	1 [0.1–2.1] *p* = .040* (0.1)^△^	
CRoM flexion (°)	Intervention	36 ± 4.6 [34–39] 35.5 [32–40]	47 ± 4.0 [45–50] 50 [44.3–50]	11 [7.8–14.2] *p* < .001** (0.9)^△△^	7 [3.9–10.2] *p* < .001^††^ (0.6)^△△^
	Control	36 ± 4.5 [34–39] 37 [31.5–40]	40 ± 4.5 [38–43] 40 [35–45]	4 [3.2–4.8] *p* < .001** (0.9)^△△^	
CRoM extension (°)	Intervention	42 ± 9.8 [37–48] 42.5 [31.5–50]	54 ± 10.2 [48–60] 56.5 [43.8–62]	12 [4.2–19.7] *p* = .012** (0.6)^△△^	10 [4.2–17.3] *p* = .003^†^ (0.5)^△^
	Control	40 ± 7.4 [36–44] 40 [33–47]	44 ± 6.3 [40–47] 45 [40–50]	4 [1.0–6.4] *p* = .009* (0.3)^△^	
CRoM lateral flexion left (°)	Intervention	44 ± 12.4 [37–51] 41 [32–56.3]	55 ± 10.1 [49–61] 56.5 [45.3–62.8]	11 [1.0–21.0] *p* = .005* (0.4)^△^	8 [1.8–14.3] *p* = .014^†^ (0.4)^△^
	Control	44 ± 6.1 [41–47] 45 [40–48]	47 ± 5.2 [44–49] 45 [45–50]	3 [1.7–4.3] *p* < .001** (0.3)^△^	
CRoM lateral flexion right (°)	Intervention	47 ± 13.6 [39–54] 47.5 [34.3–58.5]	60 ± 10.0 [54–66] 60 [54.3–70]	13 [6.1–20.4] *p* = .001* (0.5)^△^	12 [5.9–17.8] *p* < .001^††^ (0.7)^△△^
	Control	43 ± 6.4 [40–47] 44 [39–48]	48 ± 6.1 [45–51] 45 [45–52.5]	4 [2.2–6.6] *p* = .002** (0.7)^△△^	
CRoM rotation left (°)	Intervention	42 ± 7.2 [38–47] 40 [36.5–50.0]	62 ± 4.1 [60–65] 62 [59.5–66.3]	20 [16.1–23.9] *p* < .001** (0.9)^△^	12 [8.3–16.0] *p* < .001^††^ (0.8)^△^
	Control	44 ± 6.5 [41–48] 45 [38.5–51]	50 ± 6.1 [47–53] 50 [46–55]	6 [4.0–7.6] *p* < .001** (0.4)^△^	
CRoM rotation right (°)	Intervention	44 ± 3.9 [42–47] 45 [40.8–45.3]	59 ± 5.1 [56–62] 58 [55–65.3]	15 [11.7–18.5] *p* < .001** (0.9)^△^	10 [6.3–14.2] *p* < .001^††^ (0.7)^△△^
	Control	45 ± 6.5 [41–48] 42 [39.5–50]	49 ± 5.5 [46–52] 50 [44.5–54]	4 [2.4–6.5] *p* = .002^△△^ (0.7)^△△^	
PPT trapezius left (kg/cm^2^)	Intervention	2.8 ± 0.6 [2.4–2.9] 2.7 [2.4–3.4]	3.9 ± 0.5 [3.6–4.2] 3.8 [3.5–4.2]	1.1 [0.9–1.5] *p* < .001** (0.8)^△^	0.7 [0.2–1.0] *p* = .002^†^ (0.5)^△^
	Control	3.0 ± 0.6 [2.7–3.3] 3 [2.5–3.6]	3.2 ± 0.6 [2.9–3.5] 3.3 [2.8–3.7]	0.2 [0.1–0.3] *p* = .026* (0.5)^△^	
PPT trapezius right (kg/cm^2^)	Intervention	3.0 ± 0.5 [2.7–3.3] 3 [2.6–3.5]	4.1 ± 0.5 [3.8–4.4] 4.1 [3.7–4.6]	1.1 [0.8–1.4] *p* < .001** (0.7)^△^	0.7 [0.3–1.1] *p* < .001^††^ (0.6)^△△^
	Control	3.0 ± 0.3 [2.8–3.2] 2.9 [2.6–3.4]	3.4 ± 0.6 [3.1–3.7] 3.4 [2.8–3.7]	0.4 [0.2–0.6] *p* < .001** (0.5)^△^	
PPT SCM left (kg/cm^2^)	Intervention	1.4 ± 0.3 [1.2–1.5] 1.3 [1.2–1.6]	2.2 ± 0.4 [1.9–2.4] 2.3 [1.9–2.6]	0.8 [0.6–1.0] *p* < .001** (0.7)^△^	0.5 [0.1–0.8] *p* = .008^†^ (0.5)^△△^
	Control	1.5 ± 0.4 [1.3–1.8] 1.3 [1.3–1.9]	1.7 ± 0.4 [1.5–1.9] 1.5 [1.4–2.1]	0.2 [0.1–0.3] *p* < .001** (0.9)^△△^	
PPT SCM right (kg/cm^2^)	Intervention	1.5 ± 0.4 [1.3–1.7] 1.5 [1.2–1.9]	2.3 ± 0.3 [2.1–2.5] 2.4 [2.0–2.6]	0.8 [0.5–1.0] *p* < .001** (0.8)^△^	0.6 [0.3–0.8] *p* < .001^††^ (0.6)^△△^
	Control	1.4 ± 0.4 [1.2–1.6] 1.3 [1.2–1.7]	1.7 ± 0.4 [1.5–1.9] 1.5 [1.4–2.0]	0.3 [0.2–0.4] *p* < .001** (0.9)^△△^	
PPT levator scapulae left (kg/cm^2^)	Intervention	3.3 ± 0.7 [2.9–3.7] 3.5 [2.7–3.8]	4.0 ± 0.6 [3.6–4.3] 3.9 [3.7–4.4]	0.7 [0.4–0.9] *p* < .001** (0.5)^△^	0.5 [0.04–0.9] *p* = .030^†^ (0.4)^△^
	Control	3.2 ± 0.5 [2.9–3.5] 3.1 [2.8–3.7]	3.5 ± 0.5 [3.2–3.8] 3.6 [3.0–3.9]	0.3 [0.1–0.4] *p* < .001** (0.5)^△^	
PPT levator scapulae right (kg/cm^2^)	Intervention	3.1 ± 0.5 [2.9–3.4] 3.1 [2.8–3.6]	4.0 ± 0.5 [3.7–4.3] 3.8 [3.7–4.4]	0.9 [0.6–1.1] *p* < .001** (0.7)^△^	0.5 [0.06–0.9] *p* = .029^†^ (0.4)^△^
	Control	3.2 ± 0.7 [2.9–3.5] 3.0 [2.8–3.7]	3.5 ± 0.6 [3.2–3.9] 3.7 [2.9–3.9]	0.3 [0.2–0.5] *p* < .001** (0.5)^△^	

*Note*: Data are reported as mean ± SD or [95% confidence level]; and median [IQR]. Effect size with ^△^“Cohen'd” or ^△△^“Rosenthal r.”

Abbreviations: CRoM, cervical range of motion; IQR, interquartile range; JPSE, Joint Position Sense Error; NDI, Neck Disability Index; NPRS, Numeric Pain Rating Scale; PPT, pressure pain threshold; SCM, sternocleidomastoid.

* Indicates statistically significant intragroup differences (*p* < .05).

** Indicates statistically significant intragroup differences (*p* < .001).

^†^ Indicates statistically significant between‐groups differences (*p* < .05).

^††^ Indicates statistically significant between‐groups differences (*p* < .001).

Furthermore, Table 2 includes a between‐group comparison, which showed statistically significant differences (*p* < .05) and moderated effect size (d ≥ 0.6) in NPRS, NDI, PPT, and CRoM values and large effect size (d ≥ 0.8) in JPSE, in favor of the intervention group.

**FIGURE 2:**
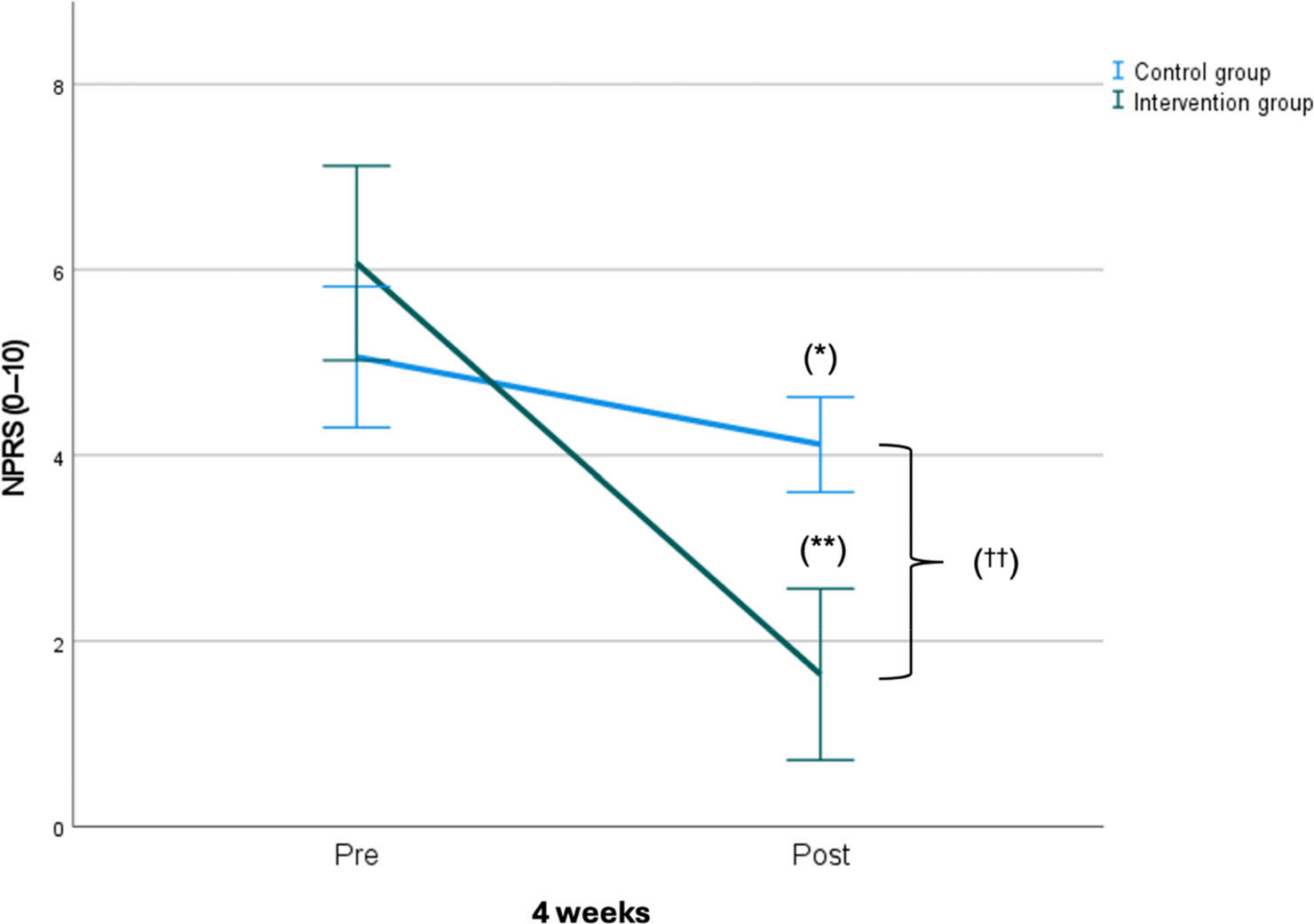
Intragroup and between‐group mean changes post intervention in NPRS. Statistically significant intragroup differences: *(*p* < .05); **(*p* < .001). Statistically significant between‐groups differences: ^†^(*p* < .05); ^††^(*p* < .001). NPRS, Numeric Pain Rating Scale.

**FIGURE 3:**
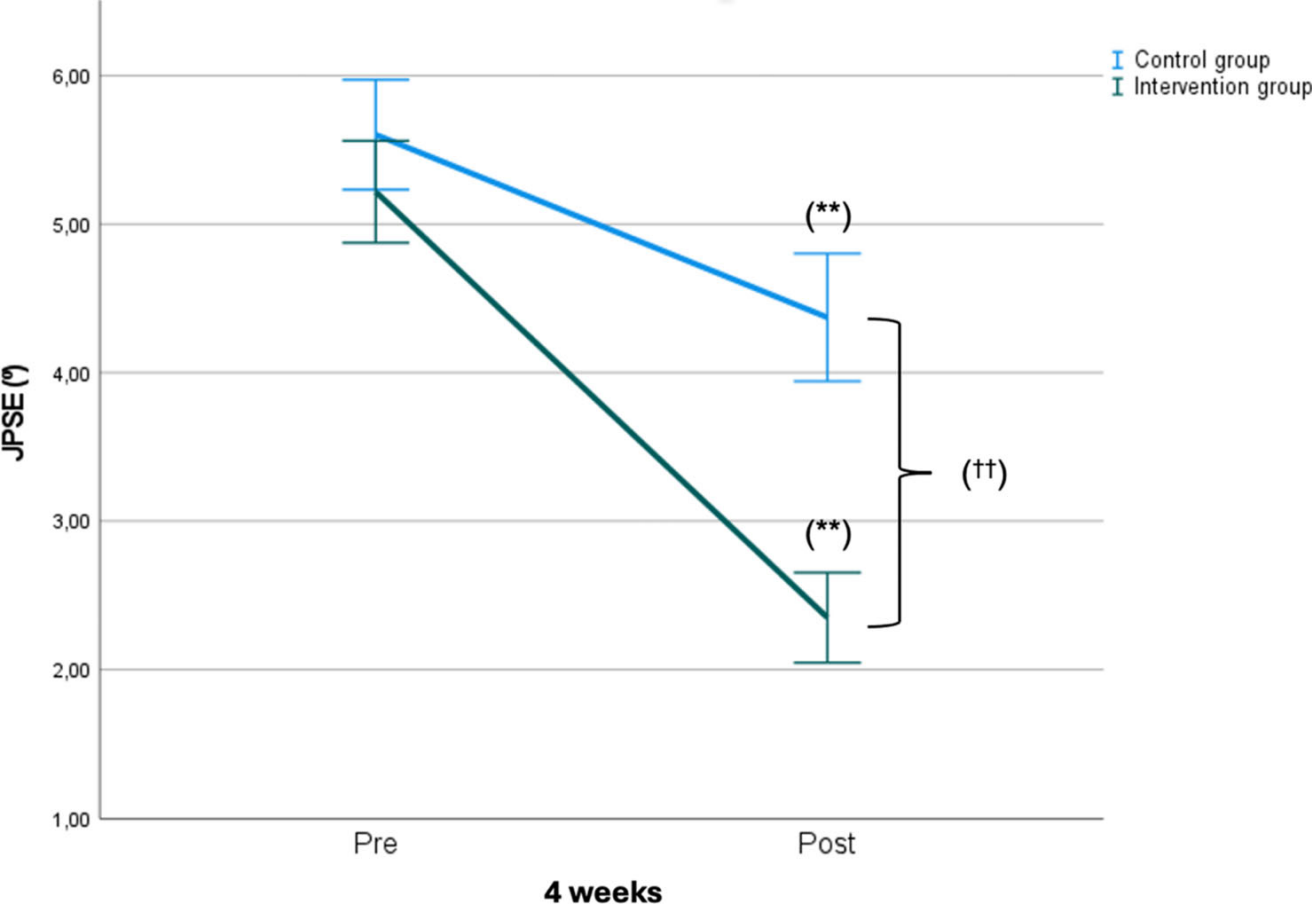
Intragroup and between‐group mean changes post intervention in JPSE. Statistically significant intragroup differences: *(*p* < .05); **(*p* < .001). Statistically significant between‐groups differences: ^†^(*p* < .05); ^††^(*p* < .001). JPSE.

Figures 2 and 3 show the mean intragroup and between‐group changes after the intervention in NPRS and JPSE, respectively.

The analysis for treatment benefit showed that 10 participants (71.4%) in the intervention group improved beyond the MCID and the MDC of the NPRS post intervention (>3 points decrease),^19^ whereas in the control group no clinically relevant improvement was observed, with no individual (0%) showing a change of >3 points. The NNT for neck pain intensity was 2 (95% CI, 2–3), with a risk ratio of 0.29 (95% CI, 0.12–0.65), an ARR of 0.71 (95% CI, 0.48–0.95), and an RRR of 0.71 (95% CI, 0.35–0.88).

As regards the JPSE, 14 participants (100%) from the intervention group scored within normality after treatment (<4.5°),^17^ compared to 7 individuals (42.2%) from the control group. The NNT for the JPSE was also 2 (95% CI, 2–3). The risk ratio was 0, with an ARR of 0.59 (95% CI, 0.35–0.82), and an RRR of 1.

No statistically significant correlation was found between the demographic characteristics of the participants and NPRS and JPSE. Therefore, there is no confounding factor in the results obtained.

## DISCUSSION

Preserving occupational health is a challenge of interdisciplinary medical rehabilitation programs in the working population. Saltychev et al.^33^ have shown the impact on work performance achieved after improvement of work‐related musculoskeletal disorders.^33^ The benefits found in the present study are consistent with the recent systematic review on the importance of detecting occupational determinants and risk factors associated with neck pain in military pilots.^34^ Consistent with clinical guidelines on the management of neck pain,^1^, ^2^, ^7^ these findings suggest that a combination of modalities yields greater effect sizes, even in high‐demand professions such as fighter pilots.

### Primary outcomes measures

Perceived pain intensity was significantly reduced in the intervention group, as well as in the control group. However, the between‐group comparison reported statistically significant differences, with higher effect sizes in favor of the intervention group. Nevertheless, the larger effect size achieved in the intervention group could be considered a more effective mediator of recovery in the intensity of FRNP experienced by fighter pilots.

These findings are consistent with previous studies that have investigated the effect of therapeutic exercise and ICT in participants with neck pain,^8^ using parameters validated in prior research.^27^, ^35^ The activation of endogenous inhibitory mechanisms by the interferential current,^8^, ^27^ Wedensky inhibition of type C nociceptive fibers and action potentials in large diameter myelinated afferent nerves^36^ and hypoalgesic effects derived from cervical exercise,^2^ could explain these results, being the first to the authors' knowledge to be applied on fighter pilots with FRNP. These results seem to clarify the lack of consensus on the combination of this current with other modalities.^36^ These effects meant that the between‐group comparison also showed statistically significant differences, with the MDC and MCID being exceeded (1.5 points and 2.6 points, respectively) in favor of the intervention group (Table 2).

In relation to JPSE, an improvement in proprioceptive acuity was also obtained, with a larger effect size in favor of the intervention group, surpassing MDC.^37^ The multimodal program may have helped in the maintenance and adjustment of craniocervical position by improving muscle activity and improving cervical muscle activation patterns.^38^ The proprioceptive system deficit observed in fighter pilots^3^ could be caused by the continuous changes of direction with high G forces, which may make the cervical postural control of fighter pilots with FRNP dependent on visual information. Therefore, the visual feedback provided by supervised exercise with the laser in these workers may be relevant.^39^

### Secondary outcomes measures

Neck disability as perceived by active military personnel has also been analyzed.^40^ Statistically significant improvements were obtained in both groups. However, between‐group comparison showed changes in favor of the intervention group with high effect size (d = 0.7). Changes in this primary outcome have been associated with the frequency with which pain persisted. The periodicity of the multimodal treatment of this study (twice a week), together with its application in the first 48 hours after the flight could possibly have influence in pain modulation and with it, in the performance of daily life activities.^41^ Further studies are needed to analyze the frequency and number of sessions needed to treat pilots with FRNP. However, these changes remained below the MDC. This limitation could be attributed to the analysis being restricted to immediate post‐treatment effects, potentially missing longer‐term changes that might have exceeded this threshold.

Previous studies with interventions similar to ours show contrasting results. Although Gallego‐Izquierdo et al.^42^ found significant reductions in perceived pain (NPRS) and disability (NDI) after proprioceptive training, the changes observed in NDI were not reported to exceed the MDC threshold. Similarly, Murray et al.^43^ found no changes in military pilots for the same variables after 20 weeks of a protocol of coordination, strength, and endurance exercise.

Baseline CRoM values in our sample were lower than age‐expected norms.^25^ This limitation is consistent with findings in fighter pilots, where CRoM limitations have been associated with occupational demands such as exposure to G‐forces and mechanical stressors.^3^, ^24^ Both groups showed statistically significant increases in CRoM, although the multimodal intervention applied in the intervention group achieved statistically superior improvements with large size effects, as did the primary outcomes. These between‐group changes are above the MDC for all variables except flexion.^23^ The desensitization of nociceptive mechanisms could be associated with a reduction of disruptive stimuli on postural control mechanisms, thus achieving a higher increase in the CRoM than that shown by Albornoz‐Cabello et al.^8^ These authors designed an intervention almost identical to the one in the present study, and the differences obtained can be explained by the following: (1) the improvement in the activation pattern of the cervical musculature derived from supervised exercise with laser guidance^44^; and (2) our sample was composed of participants with a lower average age than those who participated in the study of Albornoz‐Cabello et al.,^8^ and this may have influenced the ability to learn the proposed exercises.

Regarding PPT, our results are consistent with previous studies applied in fighter pilots^5^ with moderate or large effect sizes in the intervention group (Table 2). These improvements could be attributed to the activation of inhibitory mechanisms described herein. However, these changes did not exceed the MCID^26^ in any of the muscles analyzed.

### Clinical impact of interventions, as assessed by number needed to treat

Our findings on perceived pain (NNT = 2; RR = 0.29; RRR = 0.71; ARR = 0.71) and proprioceptive acuity (NNT = 2; RR = 0; RRR = 1; ARR = 0.59) indicate the clinical impact of both ICT and the type of exercise used on perceived intensity and cervical movement control dysfunction. Also, previous research has found that the addition of interferential therapy to therapeutic exercise is clinically more effective than therapeutic exercise alone in immediately improving neck pain (NNT = 2; RR = 0.46; RRR = 0.54; ARR = 0.52) and disability (NNT = 2; RR = 0.16; RRR = 0.84; ARR = 0.63).^8^ The improvement in cervical motor control following ELGF also appears due to the impact of this type of exercise, adding to the previously reported effectiveness.^42^, ^44^

Clinical guidelines recommend a multimodal approach in the treatment of neck pain, including exercise, manual therapy, and transcutaneous electrical stimulation.^1^, ^4^ Despite this, the observed clinical relevance supports including such procedures to reduce pain and improve proprioceptive acuity.

### Clinical implications

This innovative program enables improvements to be made in the short term, with investments in low‐cost, easy‐to‐carry and easy‐to‐operate equipment. The intervention was specifically designed to address the occupational demands of fighter pilots, aiming to mitigate deficits in motor control mechanisms associated with their professional activity. The methodology used in this program could also be adapted to other working populations with similar musculoskeletal challenges. Additionally, given its structured and reproducible design, this approach could be explored as a preventive strategy for pilots without FRNP. Further studies are necessary to explore the long‐term effects of interventions and their implications in flight safety.

### Limitations

First, only the immediate effects within 48 hours after the last session have been analyzed. Second, the sample size is limited, and the results cannot be extrapolated to other military groups. Finally, the study includes only a control group that did not receive any treatment. Future studies should include a control group applying only ICT, to determine the isolated effects of its application.

## CONCLUSIONS

Four weeks of a multimodal physiotherapy program based in ELGF with ICT seems to be effective in reducing perceived pain intensity and function outcomes in fighter pilots with flight‐related neck pain.

## DISCLOSURES

The authors declare that there is no conflict of interest.

## ETHICS STATEMENT

Ethical Research Committee of the University of Extremadura (54/2020).

## PATIENT CONSENT STATEMENT

Informed consent was obtained from all participants included in the study.

## Supporting information


**Data S1.** Supporting Information.

**Data S2.** Supporting Information.

## Data Availability

Data can be made available on request to the corresponding author.
