# Supplementary material for: Effectiveness of a multimodal physiotherapy program in fighter pilots with flight‐related neck pain: A randomized controlled trial
Source: PM R. 2025 May 23;17(11):1308–20. doi: 10.1002/pmrj.13399 (PMC12632176; doi:10.1002/pmrj.13399)
Supplement: Supplementary file 1 — Data S1. Supporting Information. [file PMRJ-17-1308-s001.docx]

**Supplementary Material 1**

**CERVICAL SUPERVISED EXERCISES WITH LASER-GUIDED FEEDBACK (ELGF)**

Cervical supervised exercises with laser-guided feedback (ELGF) is defined as a procedure of proprioceptive training based on a type of therapeutic exercise that provides external feedback to exercise, achieving an improvement in range of motion and postural control in subjects with spinal pain. According to Abdollahipour et al.^17^ and Chiviacowsky et al.^38^, the external focus promotes a kind of automatic control that reduces the awareness necessary for the exercise, favouring the retention of the learned movement pattern. Moreover, the meta-action coupling is faster, being relevant to the fighter pilot’s regular tasks. For the performance of the exercise program, “Motion Guidance Clinician Kit” (Motion Guidance LLC, Denver, CO, USA.) was used. The kit has a panel and a laser guide for the correct execution of cervical movements. The laser was positioned by means of an elastic band at the pilot’s forehead. The panel was placed 1.5 metres away from the wall. Between each series there was 10 seconds of rest. The subjects began seated on a stool and then moved to standing from the 3rd session onwards. From the 5th session onwards, the distance between the signals to be reached with the laser was increased, with the aim of increasing the range of cervical movement in the 3 planes of space. The average time to complete the entire program did not exceed 14 minutes. The program consisted of 4 exercises, which progressed in difficulty according to the tolerance achieved over the course of the sessions:

| **Exercise 1:**  Maintenance of the head position | 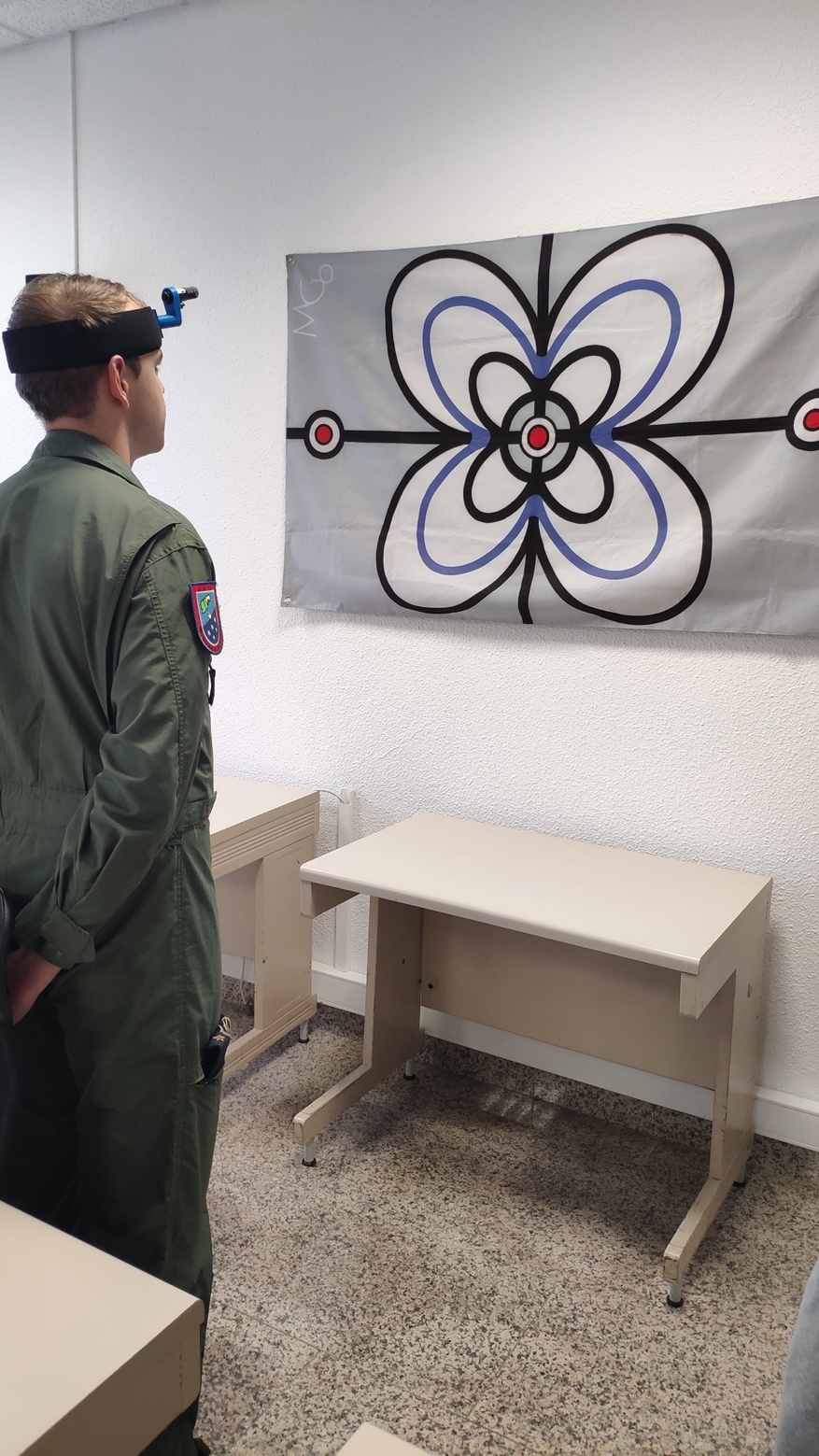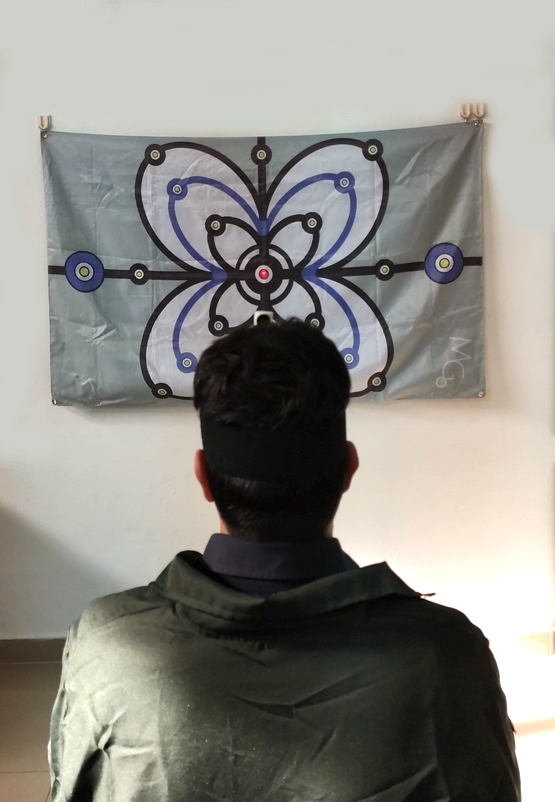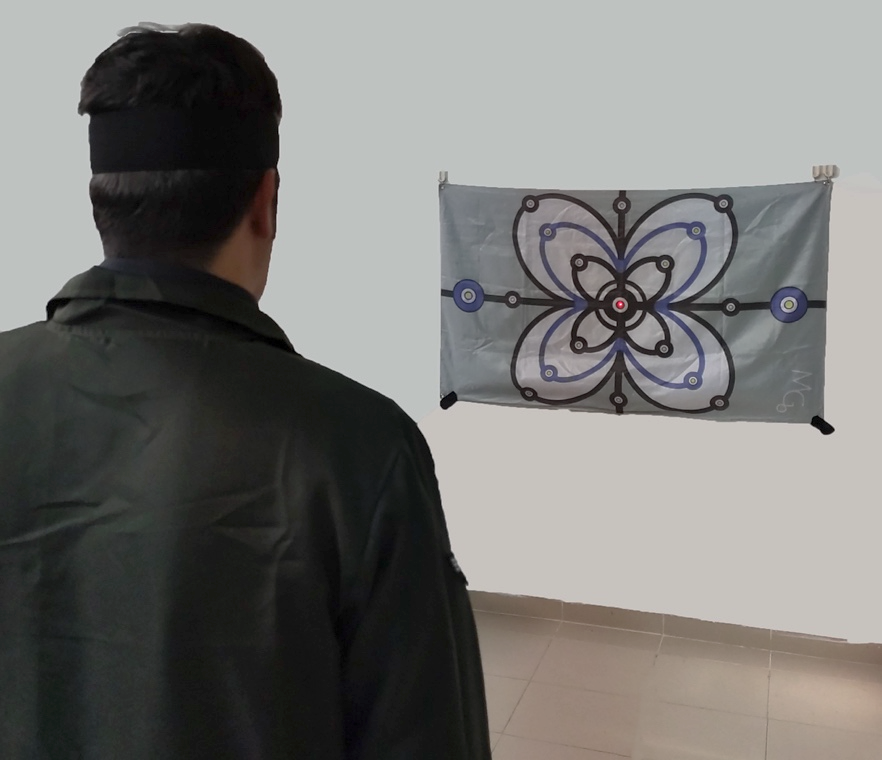 |
| --- | --- |
| **Description of the exercise:**  The laser is directed to the central point of the panel, maintaining this static position for 30 seconds, without moving off. |  |
| **Repetitions:**  4 sets of 30 seconds each. |  |
| **Progression**:  From the third session onwards, it is carried out in standing position. |  |

| **Exercise 2:**  Cervical flexion and extension. | 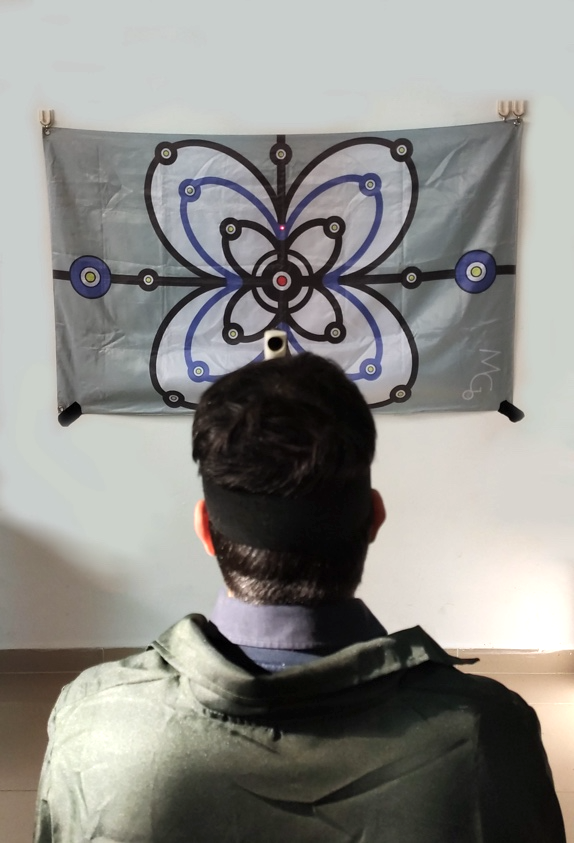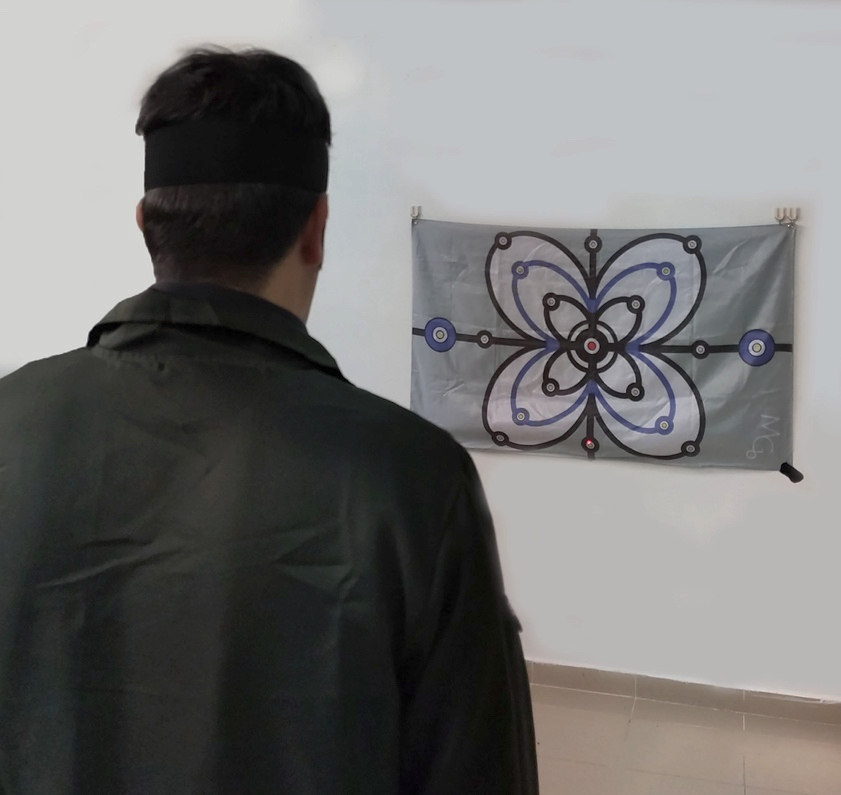 |
| --- | --- |
| **Description of the exercise:**  Starting from a cervical extension position, the laser is directed towards the upper station. The head is then bent, bringing the laser towards the lower station through the centre, without leaving the vertical line provided. Afterwards, the subject must perform a cervical extension by moving the laser vertically towards the upper station again to complete a repetition. |  |
| **Repetitions:**  4 sets of 8 repetitions each. |  |
| **Progression**:  From the third session onwards, it is conducted in standing position. From the fifth session onwards the distance between the stations is increased. |  |

| **Exercise 3:**  Right-left rotations. | 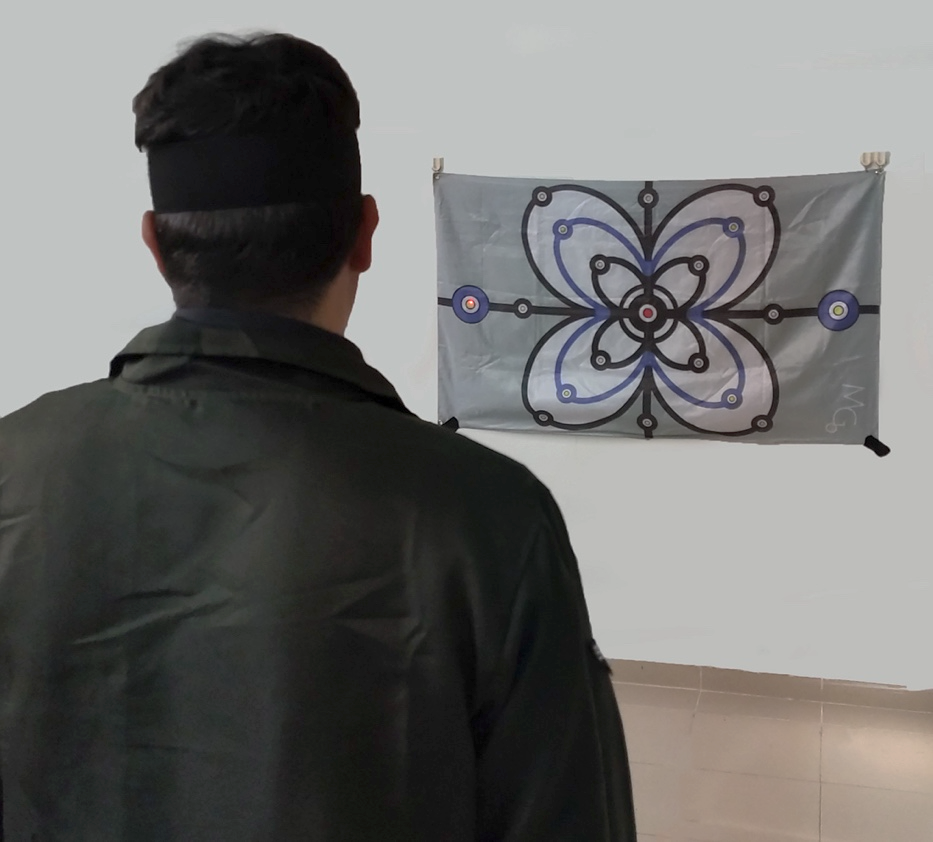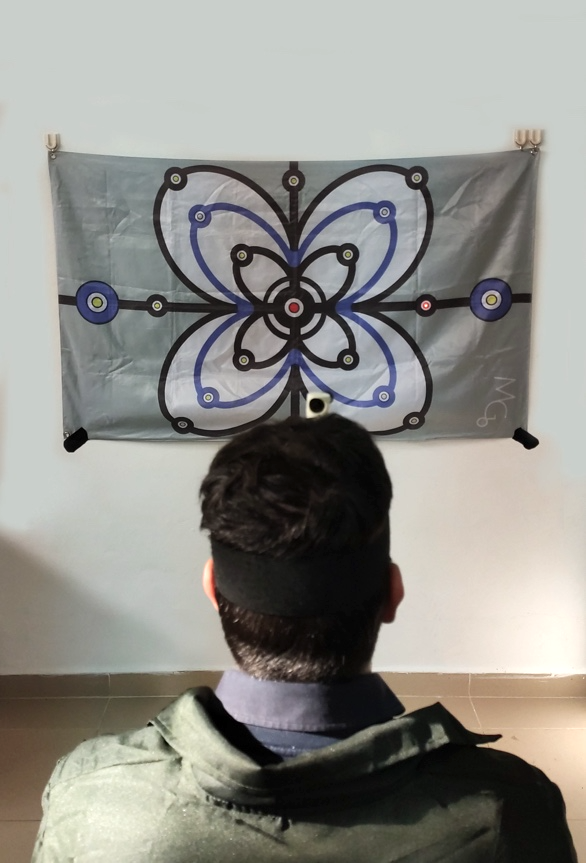 |
| --- | --- |
| **Description of the exercise:**  Starting from a right-hand rotation, the laser is directed towards the right-hand station. The head is then rotated by bringing the laser towards the left station through the centre, without leaving the horizontal line. Afterwards, the subject must perform a right-hand rotation by moving the laser horizontally towards the right station again to complete a repetition. |  |
| **Repetitions:**  4 sets of 8 repetitions each. |  |
| **Progression**:  A partir de la 3ª sesión se realiza en bipedestación. A partir de la 5ª sesión se amplía la distancia entre las señales. |  |

| **Exercise 4:**  Lateroflexions right-left. | 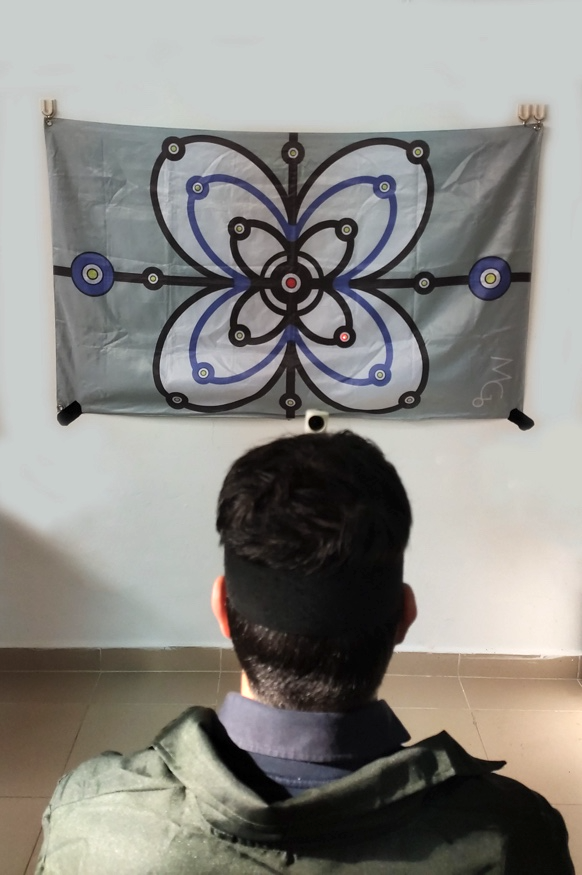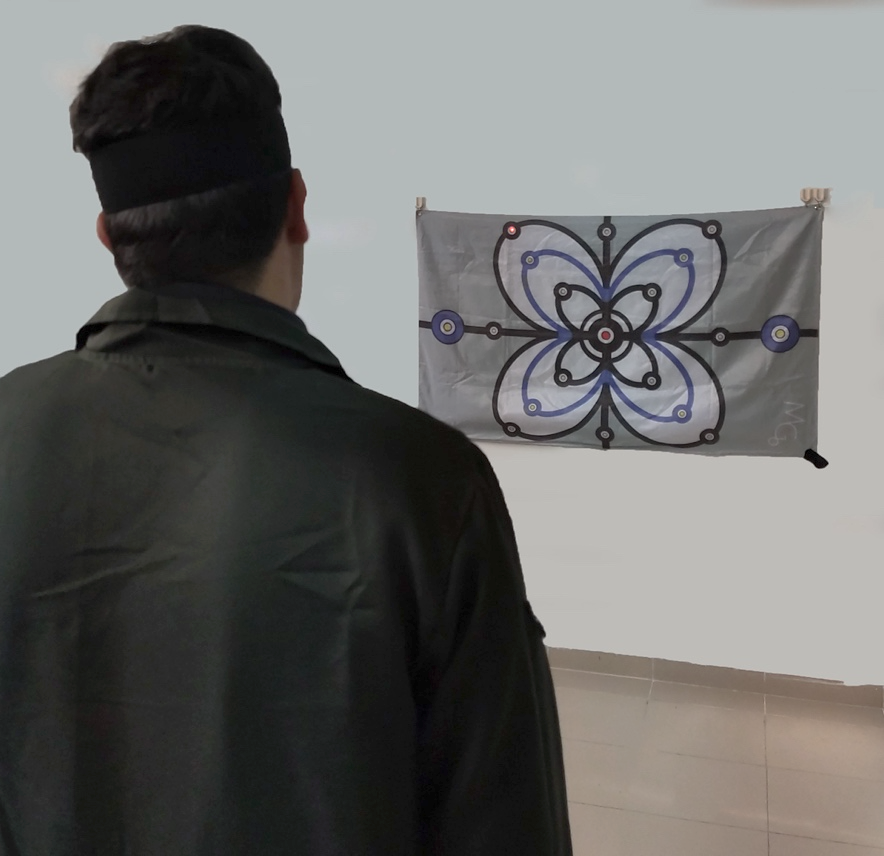 |
| --- | --- |
| **Description of the exercise:**  Starting from the central point, a right lateral flexion is performed, bringing the laser towards the two right stations. Afterwards, the subject returns to the central point and tilts laterally to the left, moving the laser to both left stations, without leaving the ellipsoidal lines. Finally, the subject returns to the central point to complete a repetition. |  |
| **Repetitions:**  4 sets of 8 repetitions each. |  |
| **Progression**:  From the third session onwards, it is conducted in standing position. From the fifth session onwards the distance between the signals is increased. |  |
