# Supplementary material for: Effectiveness of a multimodal physiotherapy program in fighter pilots with flight‐related neck pain: A randomized controlled trial
Source: PM R. 2025 May 23;17(11):1308–20. doi: 10.1002/pmrj.13399 (PMC12632176; doi:10.1002/pmrj.13399)
Supplement: Supplementary file 2 — Data S2. Supporting Information. [file PMRJ-17-1308-s002.docx]

**Supplementary Material 2**

**INTERFERENTIAL CURRENT ELECTROMASSAGE**

Interferential current electro-massage (ICE) is defined as a technique which combines simultaneously manual therapy (massage) and ICT^13^. ICE was performed in the cervical region for 15 minutes. We used a current bipolar mode, using a carrier frequency of 4000 Hz at constant voltage and an amplitude-modulated frequency of 100 Hz (Sonopuls 692®; Enraf-Nonius BV, Rotterdam, The Netherlands), was administered. The physiotherapist in charge of the intervention, in contact with the sponges (8 x 12 cm) previously moistened with hot water, performed the sequence of manual soft tissue therapy while administering the interferential current into the body through the skin^13^ by the neck, shoulder and scapular areas. The intensity was set to provide a strong and comfortable tingling, without evoking muscle twitches even though a slight vibration (fasciculation) was allowed. The electro-massage protocol lasted 15 minutes.

**INTERFERENTIAL CURRENT ELECTROMASSAGE SEQUENCE**

The sequence combined (A) superficial stroke over the neck-shoulder for 30-45 seconds; (B) deep sliding movements, alone or (C) combined with shoulder drop, for 4-5 minutes; (D) bilateral kneading of the upper trapezius (4-5 minutes); (E) slight stretching of cervical muscles (upper trapezius, sternocleidomastoid, and levator scapulae); and repetition of step (A):


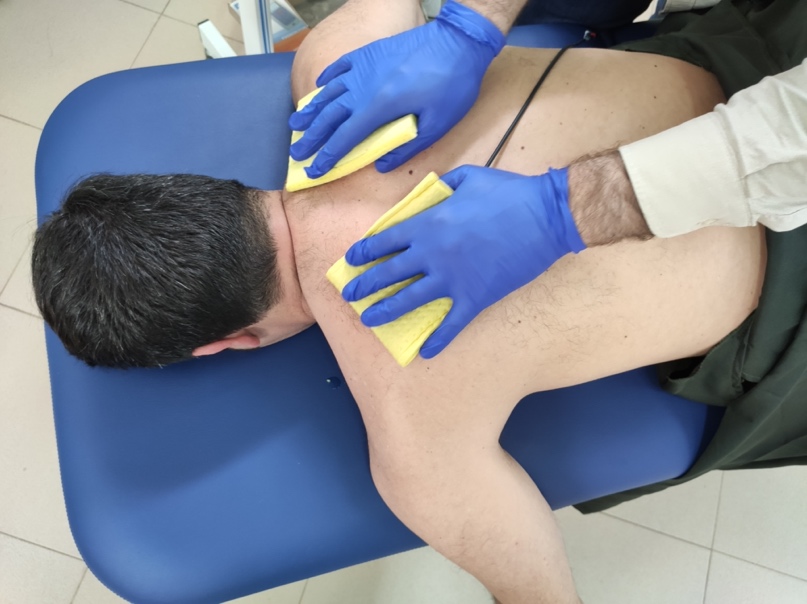

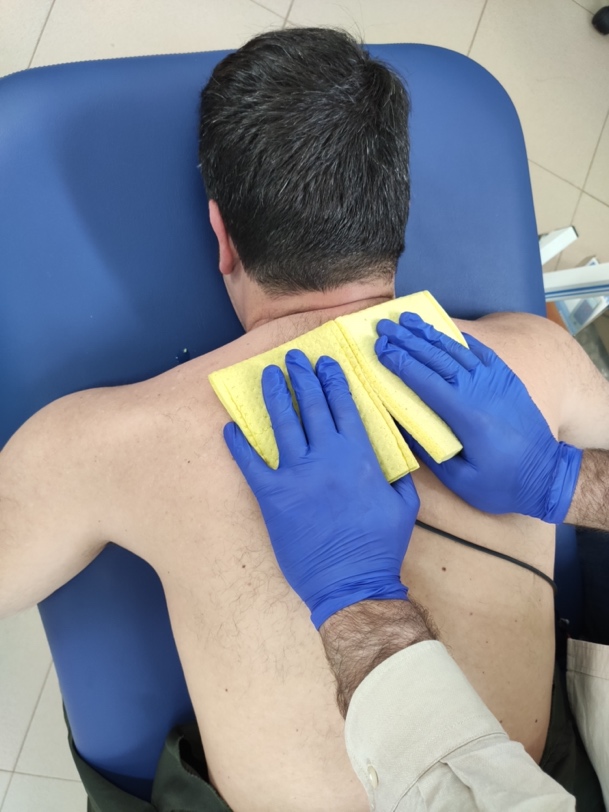


(B) Deep sliding movements (alone).

(A) Superficial stroke over the neck-shoulder.


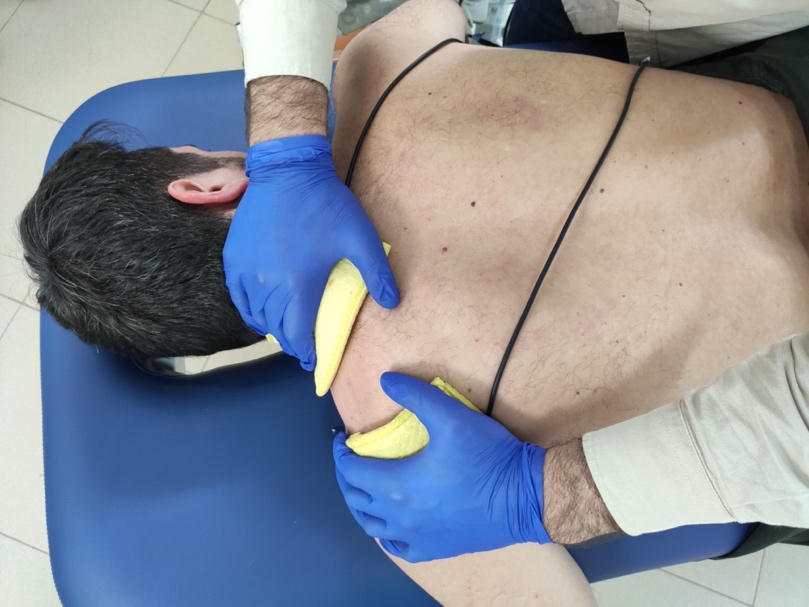

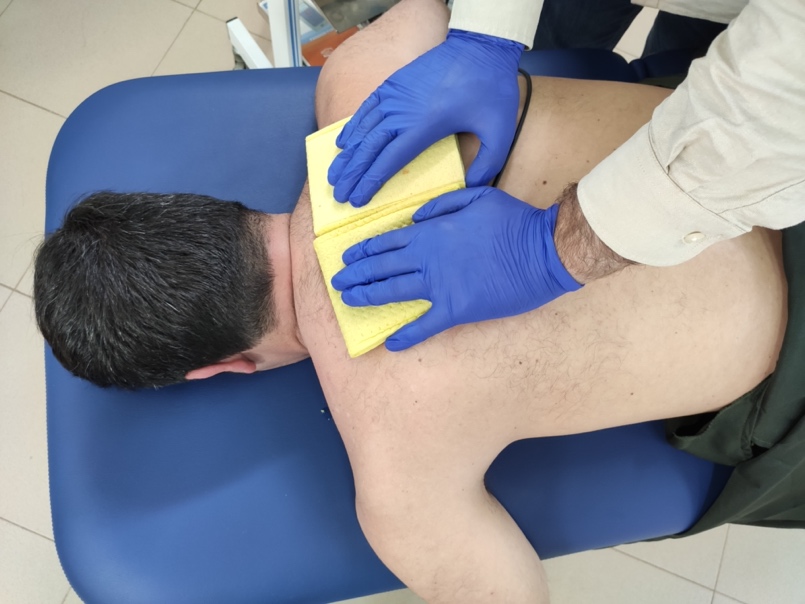

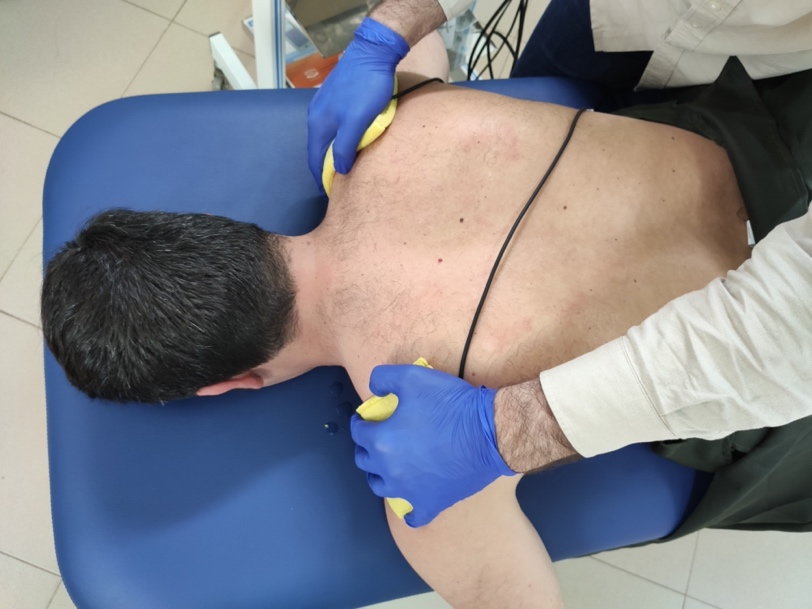

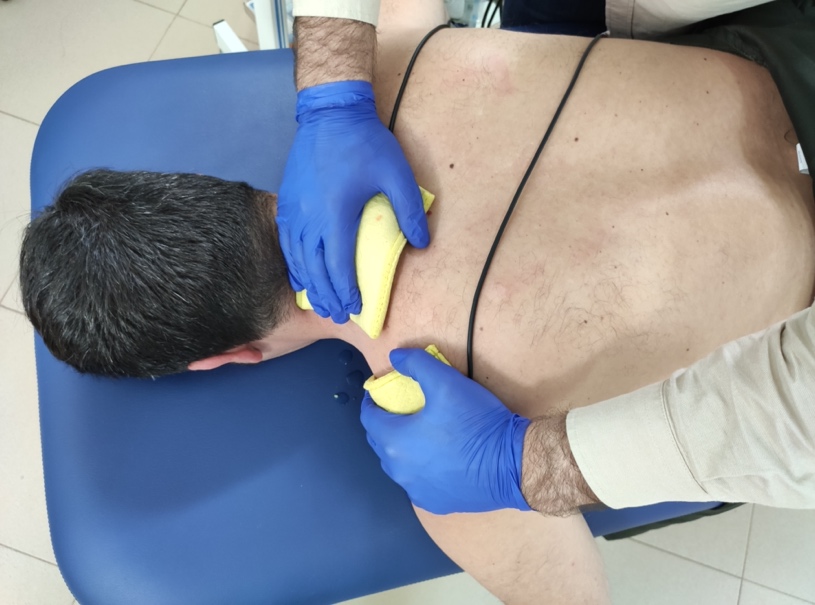


**Figure 1.** Sequence for performing Interferential Current Electromassage (ICE).

(E) Superficial stroke as in step (A).

(D) Slight stretching of cervical muscles.

(C) Deep sliding movements combined with shoulder drop.

(D) Bilateral kneading of the upper trapezius.
